# Why Are User-Generated Contents So Varied? An Explanation Based on Variety-Seeking Theory and Topic Modeling

**DOI:** 10.3389/fpsyg.2022.808785

**Published:** 2022-03-10

**Authors:** Weilin Xiang, Yongbin Ma, Dewen Liu, Sikang Zhang

**Affiliations:** ^1^College of Business, Shanghai University of Finance and Economics, Shanghai, China; ^2^Business School, Ningbo University, Ningbo, China; ^3^School of Management, Nanjing University of Posts and Telecommunications, Nanjing, China

**Keywords:** user-generated contents, variety-seeking, language/linguistic style matching, topic modeling, machine learning

## Abstract

In online communities, such as Twitter, Facebook, or Reddit, millions of pieces of contents are generated by users every day, and these user-generated contents (UGCs) show a great variety of topics discussed that make the online community vivid and attractive. However, the reasons why UGCs show great variety and how a firm can influence this variety was unknown, which had been an obstacle to understanding and managing UGCs’ variety. This study fills these two gaps based on variety-seeking theory and topic modeling, which is a technique in machine learning. We extract, quantitatively, the topic of the UGCs using topic modeling and divide UGCs into two types: single topic and multiple topics. The user’s tendency to choose the type of UGC is used to measure variety-seeking behavior. We found that users have an intrinsic preference for variety when producing UGCs; the more single topic UGCs were produced in the past, the higher the probability of producing multiple topics UGC and the lower the probability of producing single topic UGC would be in the next, and vice versa. Furthermore, we discussed the effect of language/linguistic style matching (LSM) between firm feedbacks and UGCs on users’ variety-seeking tendencies in UGCs’ production. This study makes three contributions: (1) broadening variety-seeking theory to new behavior, that is content production behavior, and the results demonstrated that people would show a variety-seeking behavior in producing UGCs. (2) a new feasible method to measure the variety of UGCs by using topic modeling to extract the topics of UGCs and then measure the variety-seeking behavior in producing UGCs by analyzing the choice between single topic and multiple topics. (3) guidance for the firm to alter LSM of feedbacks to influence the variety of UGCs.

## Introduction

In online communities, such as Twitter, Facebook, or Reddit, users can post millions of pieces of contents called user-generated contents (UGCs). UGCs convey the users’ thoughts, attitudes, or opinions; thus, UGCs vary greatly in topics that users want to discuss (Nam et al., 2017). For example, a user may produce content that discusses the topic of a certain brand’s user experience and then, produce content that includes a topic about the rival brand. However, another user might also produce the same topic or a different topic (Saha and Menezes, 2013a,b). UGCs display an abundance of topics for users to discuss, meeting the needs of varying types of consumption (McAlister and Pessemier, 1982). Moreover, the variety of UGCs’ topics reveals the various thoughts or attitudes of users, so that firms who understand these varieties can gain better market insights (Nam et al., 2017; Zhong and Schweidel, 2020).

However, such variety in UGCs’ topics brings up two unresolved questions which previous researchers had not answered yet: (1) What explains the variety in UGCs’ topics? (2) What can the firm do to change or influence the variety in UGCs’ topics? This study approaches these two questions empirically with an explanation based on variety-seeking theory and topic modeling.

Variety-seeking theory provides potential explanations for why UGCs show variety in topics. Existing literature on variety-seeking theory has demonstrated that people tend to seek variety in consuming or buying behavior; that is, if people buy one brand this time, they are likely to buy another in the next time (McAlister and Pessemier, 1982). Like consuming or buying behavior, people can also choose one type of UGCs to produce once and choose other types of UGCs to produce thereafter. Users are likely to show variety-seeking behavior in content production, which is similar to variety-seeking in consuming or buying behavior (Simonson, 1990). Therefore, according to the variety-seeking theory, the reasons why UGC is so various in topics may be that users have an intrinsic preference for variety when producing UGCs. However, producing is not consuming or buying. Previous literature on variety-seeking theory mainly discussed variety-seeking about consuming or buying behavior, whether the variety-seeking theory can be used to explain the variety of UGC topics requires empirical evidence, which is the first contribution we would make by filling this gap.

However, to conduct an empirical exploration, it is necessary to measure the variety of UGCs’ topics, but the measurement itself is a challenge. This is because traditional measurement methods are applied to structured data, that is, choices about the brand which are easy to quantify, whereas UGCs are unstructured text data, and their number could be millions, which would be difficult to measure directly using traditional measurement methods for structured data. To overcome this issue, we used topic modeling, which is a machine learning technique to extract the topics hidden in UGCs. Through topic modeling, it is possible to quantify whether users choose a single topic or multiple topics when producing UGC. The choice between a single topic and multiple topics reflects the user’s variety-seeking behavior in producing UGCs. Hence, based on topic modeling, this new measurement provides a feasible method for the quantitative analysis of variety-seeking behavior in producing UGCs. The new measurement proposed is the second contribution of this study.

Firms can impact UGCs through feedback (Piezunka and Dahlander, 2019). Previous literature has shown that the language/linguistic style matching (LSM) between firm feedback and UGCs is a powerful tool that a firm can use to influence UGCs. For example, the higher the LSM between firm feedback and UGCs, the quicker the user will produce the next UGC (Piezunka and Dahlander, 2019). However, whether LSM between firm feedback and UGCs will affect the variety of UGCs has not been investigated in previous literature. This is the third contribution we make by suggesting that a firm can adjust its feedback to influence the variety of UGCs.

The empirical results showed that users had an intrinsic preference for variety in producing UGCs, and the LSM between firm feedback and UGCs would affect variety tendency. More specifically, the more single topic UGCs the user had produced in the past, the less likely that user would produce single topic UGCs next time and also, the more likely that the user would produce multiple topics UGCs, and vice versa. We also found that the larger the LSM between firm feedback and UGCs was, the more likely the user is to produce both single topic UGCs and multiple topics UGCs, which means that the user would produce more UGCs.

The rest of this study is organized as follows. First, we review the variety-seeking literature, introducing the current progress of variety-seeking theory. Next, this study propose the hypothesis of users’ intrinsic preference for variety-seeking behavior in producing UGCs and the effect of LSM between firm feedback and UGCs on this preference. Then, to better understand variety-seeking in UGCs, the process of measuring variety-seeking behavior of UGCs production is explained. In the following section, empirical results are shown to test the hypotheses that we have put forward, along with robustness tests. Finally, contributions, implications, and limitations are discussed.

## Variety-Seeking

When a consumer makes a purchase, they often choose options that are different from those they had chosen in the past. Correspondingly, if a consumer bought a brand that had not been bought previously, we could say that he/she was seeking variety. One who switches among many different brands is considered a variety-seeker (Kahn et al., 1986). Consumers’ variety-seeking behavior is very common. For example, a consumer bought strawberry-flavored ice cream in a previous purchase, but may change to other flavors, such as vanilla ice cream, in the next purchase.

In early research about variety-seeking, scholars noticed that people have an intrinsic preference for variety and show a tendency to seek variety in consumption. For example, according to Brickman and D’Amato (1975) and Faison (1977), if consumers continue to consume the same products, the utility obtained diminishes marginally, regardless of whether these products are unfamiliar or even familiar to consumers. To maximize the utility from product consumption, consumers will switch among products, which is manifested as variety-seeking behavior. Hirschman (1980) found that consumers would pursue novelty, and various products can be the potential source of novelty. Hence, consumers will desire unfamiliar things and show variety-seeking inclination. McAlister and Pessemier (1982) systematically summarized variety-seeking behavior and highlighted that in addition to pursuing novelty, consumers have an intrinsic desire for change, and eventually they would show variety-seeking behavior in consuming or buying. Kahn (1995) pointed out that people have an inner need for stimulation, which could be satisfied *via* variety-seeking in consuming or buying.

Subsequently, scholars have investigated and made progress on what might affect variety-seeking behavior in consuming or buying. Generally speaking, three factors were studied: product characteristics, external factors, and other factors.

First, product characteristics were demonstrated to affect variety-seeking behavior. For example, Chuang et al. (2013) found that if the word-of-mouth of an option was better, consumers tended to show variety-seeking behaviors. Song et al. (2019) showed that consumers would not always seek variety with a fixed pattern. One would continuously learn from the product during the process of consumption and update his/her preference for the final choice. Lee et al. (2020) studied the cross-promotion of mobile apps and found that if the similarity between the target app and the app which the user used before is higher, the number of app downloads, the number of apps opened after download, and the running time after the download would increase first and then decrease, which was shown to be an inverted U relationship, that is, users did not like unfamiliar apps nor too familiar apps; therefore, users would show variety-seeking in apps download.

Second, some scholars have noted that external factors influence variety-seeking in consuming or buying. For example, Yoon and Kim (2018) exploited experiments which showed that if a consumer felt that their economic mobility and socioeconomic status were poor, he/she will feel a lack of control, and the lack of control will prompt he/she to seek variety in consuming or buying to regain control.

Third, other factors also influence variety-seeking in consuming or buying, such as personal characteristics, biological rhythms, and sleepiness. For example, Fernandes and Mandel (2014) pointed out that politically conservative people are more inclined to abide by social norms. They observed that in places like the United States and Europe, maintaining variety is a social norm and people are more inclined to show variety-seeking behavior. Using millions of shopping data, Gullo et al. (2019) found that at different times of the day, consumers show different variety-seeking tendencies. This is because the body temperature is lower in the morning, along with a low level of arousal, which leads to a low need for variety. Huang et al. (2019) showed that if consumers feel sleepy, they will require arousal, and variety can provide stimulation; then, sleepy consumers will show variety-seeking behaviors in consuming or buying.

In summary, the existing literature on variety-seeking theory is very rich, and much research has been conducted to reveal why people show variety-seeking in consuming or buying. However, people could also show variety-seeking in producing, such that they could choose a certain type of topic to produce UGCs, and choose another type of topic to produce UGCs subsequently. However, existing variety theory research has not focused on variety-seeking in producing, which leaves an important gap that we try to fill.

## Hypotheses

### Intrinsic Preference for Variety-Seeking in User-Generated Contents

People have an intrinsic preference for variety, and previous literature has demonstrated this from three perspectives: the optimal level of stimulation, uncertainty, and novelty.

First, people have an intrinsic preference for variety to obtain the optimal level of stimulation. Faison (1977) pointed out from the perspective of the optimal level of stimulation that people will switch among a set of choices to obtain the greatest level of stimulation from consumption, and if a product is repeatedly consumed, consumers feel disgusted. To eliminate the sense of disgust, people tend to seek variety in consuming or buying. Similarly, it is very likely that if a user repeatedly produces UGCs with the same type of topic, they will not get the greatest level of stimulation. Therefore, when producing UGCs, the user tends to show variety-seeking and switch among different types of topics to maximize the stimulation of UGCs.

Second, people have an intrinsic preference for variety to reduce uncertainty. Pessemier (1978) pointed out that to reduce uncertainties and risks about products, consumers will try to collect and update the true information of each product, which results in the pursuit of variety-seeking in consuming or buying. Similarly, the production of UGCs may also face uncertainties and risks. Therefore, to reduce uncertainties and risks about different type of topics when producing UGCs, users will try to collect and update the true information about different type of topics, which results in switching among different types of UGCs and pursuing variety-seeking in producing UGCs. For example, users do not know whether the feedback he/she will get for his/her UGCs is positive or negative. To reduce such uncertainties, users could collect more information about whether he/she can get positive or negative feedback for each type of topic by switching among different types of topics, which is the pursuit of variety-seeking in producing UGCs.

Third, consumers have an intrinsic preference for variety to satisfy the natural demand for novelty (Hirschman, 1980; McAlister and Pessemier, 1982). Consumers try to consume or buy different products and obtain novelty from them. This behavior is manifested as a tendency to seek variety in consuming or buying. Similarly, to make UGCs more popular, users will try their best to maintain the novelty of UGCs, which results in switching among different types of topics to pursue variety-seeking in producing UGCs.

Overall, people have an intrinsic preference for variety. It is reasonable to believe that users will seek variety in producing UGCs. Because the production of content is completely and unlimitedly determined by the user, it may contain many topics. To simplify the analysis, topics of UGCs were divided into two categories: single topic UGCs that contains only one topic, and multiple topics UGCs that contains no less than two topics. The preference for variety in producing UGCs is reflected in the switch between single topics and multiple topics in producing UGCs.

**Hypothesis 1:** The more single topic UGCs a user has produced, the more likely it will be for the user to produce multiple topics UGCs in the next (H1a), and the less likely it will be to produce a single topic UGCs afterward (H1b).

**Hypothesis 2:** The more multiple topics UGCs a user has produced, the less likely it is for the user to produce multiple topics UGCs next (H2a), and the more likely it is to produce a single topic UGCs afterward (H2b).

At first sight, H1b/H2b might seem redundant because if a UGC is not a single topic, it has to be multiple, and H1a/H2a is sufficient. However, a user can produce more than one UGC next; some UGCs might be single topic, and others might be multiple topics. Therefore, it was necessary to consider both H1b and H2b.

### The Effect of the Linguistic Style Matching of Firm Feedback on Variety-Seeking in User-Generated Contents

Piezunka and Dahlander (2019) highlights that the LSM of firm feedback has a positive effect on the number of UGCs. So, it may have a positive effect on the probability of producing single topic UGCs or the probability of producing multiple topics UGCs, or both. In comparison with just positively increasing the probability of producing single topic UGCs or the probability of producing multiple topics UGCs, if the LSM of the feedback promote users to produce more single topic UGCs and more multiple topics UGCs, users will show more variety-seeking. Hence, the question we try to answer here is whether the LSM of firm feedback might influence the users’ variety-seeking in producing UGCs by increasing the probability of producing single topic UGCs and multiple topics UGCs simultaneously.

The LSM literature has provided useful insights into this question. Niederhoffer and Pennebaker (2002) found that, in the communication of two parties, if the participants of any party are more willing to participate in the conversation, then the LSM between the two parties will be higher, but if the two parties in the conversation are not very willing to participate in the conversation, then LSM between the two parties will be lower. This finding implies that LSM could be used to analyze the relationship in communication, and studies from different fields have confirmed that it could be a powerful tool. For example, people prefer to date someone with a similar language style, and if the LSM of both parties is higher on subsequent dates, the relationship between the two parties will be longer (Ireland et al., 2011); the higher the LSM between the interviewer and the interviewee, the more likely the interviewer is to communicate with the interviewer in an empathetic way (Meinecke and Kauffeld, 2019).

Therefore, if the LSM between the firm feedback and UGCs is higher, it conveys the information to the user that the firm is willing to communicate with the user after reading UGCs. In addition, in psychology, the higher the LSM, the shorter the social distance between the two parties will be, and the more consistent the mutual identity between the two parties (Giles and Ogay, 2007; Ireland and Pennebaker, 2010). Then, when the LSM between the firm feedbacks and UGCs is greater, the user is more willing to produce both single topic UGCs and multiple topics UGCs, and the user will show more variety-seeking in comparison with just producing single topic UGCs or more multiple topics UGCs, which implies that the LSM of firm feedback will influence the variety-seeking of users in producing UGCs, so we put forward the following hypotheses:

**Hypothesis 3a:** If the LSM between firm feedback and UGCs produced by a user is higher, the user will be more likely to produce single topic UGCs next.
**Hypothesis 3b:** If the LSM between firm feedbacks and UGCs produced by a user is higher, the user will be more likely to produce multiple topics UGCs next.

Again, we need to highlight that Hypotheses 3a and Hypothesis 3b are neither contradictory or redundant. This is because the user could choose not to produce UGC in the future or choose to produce many UGCs. Therefore, there may be a situation in which the probability of producing a single topic and multiple topics UGCs increases simultaneously. Then, if Hypotheses 3a and Hypothesis 3b are established, simultaneously, users will produce more UGCs; that is, the higher the LSM between firm feedbacks and UGCs, the more UGCs users may produce. Thus, we propose the following hypothesis:

**Hypothesis 4:** If the LSM between firm feedbacks and UGCs produced by a user is higher, the user will produce more UGCs next.

## Materials and Methods

### Data

The data in this study were collected from the posts in the new functions section under MIUI Forum^1^, which is an online community established by Xiaomi in 2010 for users to discuss Xiaomi’s mobile operating system MIUI. In the new functions section, users can post discussion issues or make suggestions about MIUI.

This study collected: (1) the data of posts, including the data of user-produced content, the post producer’s identity (which is used to determine if they are firm employees who represent the firm), post time, and post content; (2) the feedback data of posts, including the feedback producer’s identity, feedback time, and feedback content. According to the identity of the feedback producer, this paper divides the post feedbacks into other user feedbacks and firm feedbacks.

Considering that the MIUI forum was established in 2010 and experienced rapid growth in the early stage of the forum, the time range of data is Jan. 1, 2013, to Dec. 31, 2014. However, Xiaomi has made two major upgrades to its MIUI system during this period, MIUI V5 was released on Apr. 9, 2013, and MIUI 6 was released on Aug. 16, 2014, which is similar to the iphone’s upgrade from IOS 5 to IOS 6. To avoid potential impacts before and after the release of the new system, this study finally selected data 2 months after the release of MIUI V5 to 2 months before the release of MIUI6, a total of 373 days, corresponding time ranges from Jun. 9, 2013, to Jun. 16, 2014. Finally, because the variety-seeking behaviors we focus on belong to massive users but not the firm, and there are only 58 posts produced by the firm, which is a small number, we removed these 58 posts produced by the firm and their corresponding feedback.

The final data sample includes 9,681 users and 17,401 posts, of which 10,491 are multiple topics posts and 6,910 are single topic posts. In this paper, the month is taken as the minimum time unit to record the posts of each user and the feedback received by each user each month. It should be noted that this paper used gensim, an open-source third-party library in Python introduced by Hannigan et al. (2019), to analyze the topics of the text. Although Hannigan et al. (2019) summarized that there are many software, such as R language/Java that could extract text topics, we used gensim^2^.

### Measurement of Variety-Seeking in User-Generated Content: Topic Modeling

Previous research has put forward many methods to measure the variety-seeking in consuming or buying products; one of the most commonly used measurements is to measure the number of non-repeating brands in a certain number of products (Levav and Zhu, 2009; Huang et al., 2019). But the number of products is structured data and UGCs are unstructured data and often in the millions, even hundreds of millions, which means the previous measurement of variety-seeking are not applicable. To deal with such unstructured and enormous data, this paper adopts the topic modeling proposed by Blei et al. (2003).

Topic modeling can be used to extract the main topics of large amounts of UGCs, and this method has been already widely used. For example, Nam et al. (2017) analyzed the label topics posted by users on social media and found that changes in topics can reflect the dynamic changes of the brand perception of users; Zhong and Schweidel (2020) found that before and after the outbreak of Volkswagen’s exhaust gas fraud in 2015, the topics in UGCs related to Volkswagen on social media changed significantly. Before the outbreak of Volkswagen’s exhaust gas fraud, topics in UGCs were mainly about the user experience and maintenance of Volkswagen cars, but after the outbreak of Volkswagen’s exhaust gas fraud, it was mainly about the details of the fraud and details of the EPA’s lawsuit against Volkswagen.

Since topics in UGCs reflect their meaning, users choose a single topic or multiple topics. This choice is an ideal measurement of variety-seeking in producing UGCs. Therefore, we used topic modeling to analyze variety-seeking in producing UGCs.

To clarify how the topic modeling works in an easy-to-understand way, we will explain the topic modeling concisely, for more details about the derivation, see the Supplementary Material of this paper.

Using topic modeling, we can get a topic combination hidden in UGC, that is, ($p_{1}^{*}\,\,t\,o\,p\,i\,c_{1},p_{2}^{*}\,\,t\,o\,p\,i\,c_{2},$…, $p_{k}^{*}\,\,t\,o\,p\,i\,c_{k}$), where, *p*_*k*_ is the ratio or weight of *topic*_*k*_ in UGC. $p_{k}^{*}\,\,t\,o\,p\,i\,c_{k}$ means that *topic*_*k*_ account for *p*_*k*_ in UGC. Thus, the larger *p*_*k*_ is, the more likely UGC is from *topic*_*k*_. What we need to highlight is that the sum of all *p*s is 1 and *p*_*k*_ could be minimum to 0 or maximum to 1. There is only *topic*_*k*_ in UGC as long as all *p*s except *p*_*k*_ is 0.

In the topic combination, if there is a *p* greater than threshold 0.5, the UGC is regarded as a single topic; otherwise, the UGC is regarded as multiple topics. Therefore, users’ choice tendency between single topic and multiple topics UGC is the variety-seeking tendency in the production of UGC. In addition, this study changed the threshold value in the robustness test to prove that the results are not sensitive to the particular threshold value.

**FIGURE 1:**
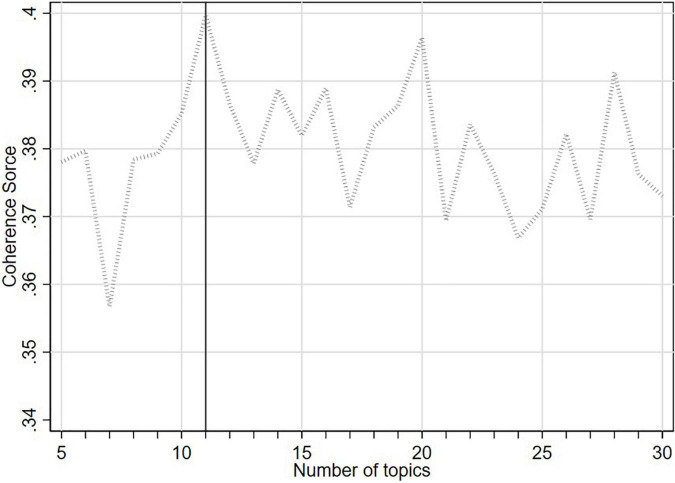
The number of topics and the corresponding *Coherence Score*.

For topic modeling, we need to specify the number of topics in advance (Blei et al., 2003), because the MIUI forum data used in this paper is not like the news that has a definite and obvious number of topics, such as entertainment, finance, politics, etc. In the existing literature, there are two indexes for determining the optimal number of topics, namely *Perplexity Score* and *Topic Coherence Score*. Lozano et al. (2017), Ye et al. (2020), and Zhong and Schweidel (2020) used *Perplexity Score* to determine the optimal number of topics. The principle of *Perplexity Score* is that, the lower the *Perplexity Score*, the better the prediction power of the constructed model. However, Newman et al. (2010) pointed out that *Perplexity Score* does not take into account the interpretability of the topics, and *Topic Coherence Score* performs better in interpretability. The principle of *Topic Coherence Score* is that, the higher the *Topic Coherence Score*, the better it will be in interpretability, indicating that there are enough differences between topics, which is also more consistent with the reality that there should be enough differences between topics. Therefore, in this study, the *Topic Coherence Score* is selected as the index to determine the optimal number of topics, and the optimal number of topics from 5 to 30 is tested. The analysis results are shown in Figure 1.

According to Figure 1, when the number of topics is 11, the *Topic Coherence Score* is the highest, and the number of topics is relatively small. Therefore, the optimal number of topics is determined as 11, so that all potential topics can be identified from the users’ posts as much as possible, and too many topics will not be added to induce difficulties in the interpretation of topics.

To explain the meaning of 11 topics, this study has selected the top 20 words that appear most frequently under each topic. It should be noted that topic modeling can only determine the number of topics and word distribution under each topic, and the final meaning of each topic needs to be explained by manual work (Zhong and Schweidel, 2020). Therefore, to understand the results of topic modeling, these 11 topics are explained in this article. Since this study is not concerned with how to interpret each topic, but with the variety-seeking behavior between single topic and multiple topics, the interpretation does not affect the subsequent analysis. Table 1 shows the 20 words with the highest frequency in the first three topics. For the complete word frequency table of the 11 topics, please see Supplementary Table 1

**TABLE 1:** The top 20 words in the first three topics.

Topic	The top 20 words	Explanation on topic
Topic 1	*[“functions,” “messages,” “recommendations,” “in,” “hope,” “has,” “add,” “process,” “reply,” “software,” “delete,” “desktop,” “very,” “application,” “block,” “no,” “notify,” “set up,” “join,” “all”]*	MIUI SMS function related
Topic 2	*[“topic,” “application,” “MIUI,” “Xiaomi,” “all,” “no,” “system,” “user,” “updated,” “very,” “function,” “good,” “hope,” “shop,” “mobile phone,” “development,” “said,” “now,” “modules,” “miui”]*	MIUI topic related
Topic 3	*[“flow,” “show,” “card,” “network,” “weather,” “recommendations,” “when,” “3G,” “mobile phone,” “Mobile and Unicom,” “no,” “assistant,” “use,” “color,” “all,” “operator,” “package,” “hope,” “charging”]*	Mobile plans, etc.

To further illustrate the representation of topics for UGCs, this paper selected some posts with the highest probability of occurrence under each topic. Table 2 shows the posts that are relatively close to the first three topics. In fact, each post has a probability of belonging to 11 topics, and the table picks the topics with the highest probability to report. The fourth column of the table is the probability that a post belongs to that topic. For the complete table of 11 topics, see Supplementary Table 2.

**TABLE 2:** Examples of posts corresponding to the first three topics.

Topic	Explanation on topic	One original post	Probability of the topic
Topic 1	MIUI SMS function related	.“*I use the contact group function in MIUI, which is very helpful for contact classification. But now I have a hope. Such as my group has: | “Shanghai” | “Harbin” | | “students” “teacher” | “high school” | | “university”……”*	0.982
Topic 2	MIUI topic related	.“*Damn it, now the default font is applied to all topics. The result will be restored to the default font. Reset the font every time you apply the topic. Except for the font. A lot of times. Topics are unlikely to be perfect in every module. Every module that can’t be done is liked by users. So it’s recommended to apply topics….”*	0.989
Topic 3	Mobile plans, etc.	.“*Send between 2082 and 10010, received the following reply | | | as of October 7, your meal allowance for the month information is as follows: … Now the 2G card of Ningxia Unicom cannot be automatically corrected with traffic. I hope it can be repaired.”*	0.968

*“| “ in original post means newline and does not influence any conclusion in this manuscript.*

In conclusion, it is reasonable to believe that the variety-seeking behavior of users in UGCs production can be measured based on the topic probability of the users’ posts. Again, the definition of single topic and multiple topics posts refer to the probability threshold of 0.5. Specifically, a post is considered a single topic post if the probability of it coming from any topic is greater than 0.5, and a multiple topic post otherwise. For example, if the probability of coming from a certain topic is more than 0.5, it indicates that there is a high proportion of the content of the post discussed around this topic, so it is reasonable to regard the content of the post as spread around the topic, which can also be regarded as a single topic of the post. Nevertheless, different thresholds will be replaced in the robustness test to prove the robustness of the conclusion.

### Measurement of Linguistic Style Matching

To calculate the language style matching (LSM) between firm feedbacks and UGCs, it is necessary to categorize the part of speech of the words in text. For example, Niederhoffer and Pennebaker (2002), when studying LSM, considered the degree of word matching between the affective and cognitive words. However, since Ireland and Pennebaker (2010), more and more literature only considered the matching degree of function words between texts when studying LSM. This is because non-functional words (e.g., nouns, adjectives) reflect the content of the text, while functional words (e.g., pronouns, quantifiers) reflect the style of the text (Chung and Pennebaker, 2007; Tausczik and Pennebaker, 2010). LSM refers to the matching of the two parties in the language style, so it is more reasonable to use function words.

Specifically, Ireland and Pennebaker (2010) used nine types of function words to study LSM, namely, personal pronouns (e.g., I, you), non-personal pronouns (e.g., this, it), articles (e.g., A, the), auxiliary verbs (e.g., am, have), and high-frequency adverbs (e.g., Very, well), prepositions (e.g., in, around), conjunctions (e.g., but, while), negative words (e.g., not, no), quantifiers (e.g., many, few). However, Chinese has its own function words system. Therefore, based on the ideas of Ireland and Pennebaker (2010) and combined with the *Modern Chinese Function Word Dictionary*^3^, we divide function words into seven types: auxiliary words, adverbs, prepositions, conjunctions, pronouns, positional words, and modal words. The text word segmentation and word part of speech recognition is completed by the third-party Python library jieba, which is a common practice to analyze Chinese (Zhou et al., 2019)^4^.

Referring to Ludwig et al. (2014), taking two texts A and B as examples, the calculation process of LSM is as follows. First, the proportion of seven function words of text A and B is calculated. For example, the proportion of auxiliary words of text A and text B is:


(1) $${\mathrm{FWC}}_{A,\mathrm{auxiliary}}=\frac{\mathrm{Number}\,\mathrm{of}\,\mathrm{auxiliary}\,\mathrm{words}\,\mathrm{in}\,\mathrm{text}\,A}{\mathrm{number}\,\mathrm{of}\,\mathrm{words}\,\mathrm{in}\,\mathrm{text}\,A}$$

(2) $${\mathrm{FWC}}_{B,\mathrm{auxiliary}}=\frac{\mathrm{Number}\,\mathrm{of}\,\mathrm{auxiliary}\,\mathrm{words}\,\mathrm{in}\,\mathrm{text}\,B}{\mathrm{number}\,\mathrm{of}\,\mathrm{words}\,\mathrm{in}\,\mathrm{text}\,B}$$

Then, the LSM of text A and text B on these seven function words is calculated. For example, the LSM of text A and text B on auxiliary words is:


(3) $${\mathrm{LSM}}_{\mathrm{AB},\mathrm{auxiliary}}=1-\frac{\left|{\mathrm{FWC}}_{A,\mathrm{auxiliary}}-{\mathrm{FWC}}_{B,\mathrm{auxiliary}}\right|}{{\mathrm{FWC}}_{A,\mathrm{auxiliary}}+{\mathrm{FWC}}_{B,\mathrm{auxiliary}}+0.0001}$$

The purpose of adding 0.0001 to the denominator is to prevent the denominator from being 0. In addition, it can be seen from Equation 3 that the value of LSM is between 0 and 1. The higher the LSM is, the more matching the language style of text A and text B is.

Finally, the LSM between text A and text B is the average of LSM on these seven function words, namely:


(4) $${\mathrm{LSM}}_{\mathrm{AB}}=\frac{1}{7}({\mathrm{LSM}}_{\mathrm{AB},\mathrm{auxiliary}}+{\mathrm{LSM}}_{\mathrm{AB},\mathrm{adverbs}}+\ldots +{\mathrm{LSM}}_{\mathrm{AB},\mathrm{modal}})$$

Referring to the processing method of Ludwig et al. (2014), for two or more UGCs produced in the same month, these UGCs are regarded as belonging to one UGC but from different paragraphs of the UGC. Similarly, based on distinguishing the feedbacks from the firm or other users, the LSM between the firm feedbacks and the user’s UGCs, and the LSM between feedbacks from other users and the user’s UGCs are both processed in this way.

### Model

Referring to the method of Bayes (2013), this paper uses the panel Logistics model to verify hypothesis 1 to hypothesis 3. The model is as follows.


(5) $$\mathrm{Prob}\left(\mathrm{dmy}\_{\mathrm{single}}_{i,t}=1\right)=\frac{\exp\,\left(X_{i,t-1}\,W+\varphi_{i,t}\right)}{1+\exp\,\left(X_{i,t-1}\,W+\varphi_{i,t}\right)}$$

(6) $$\mathrm{Prob}\left(\mathrm{dmy}\_{\mathrm{multi}}_{i,t}=1\right)=\frac{\exp\,\left(X_{i,t-1}\,W+\varphi_{i,t}\right)}{1+\exp\,\left(X_{i,t-1}\,W+\varphi_{i,t}\right)}$$

In Equations 5, 6, *i* represents the individual user, *t* represents the month, *dmy*_*single*_*i*,*t*_ and *dmy*_*multi*_*i*,*t*_ are dummy variables. The variable *dmy*_*single*_*i*,*t*_ indicates whether the user has posted at least one post with a single topic in the month *t*, 1 indicates yes, and 0 indicates other. Similarly,*dmy*_*multi*_*i*,*t*_ is defined as whether user *i* will produce at least one multiple topics post in month *t*. The advantage of using dummy variables is to take the possibility that users do not produce into account. *X*_*i*,*t*−1_ includes core variable and control variable, φ_*i*,*t*_ includes individual fixed effect, time fixed effect, and random disturbance term. Except for φ_*i*,*t*_, the definition of each variable is shown in Table 3.

**TABLE 3:** The definition of variables.

Variable	Definition
*dmy*_*single*_*i*,*t*_	Dummy variable, 1 means that user *i* has produced at least one single topic post in month *t*, otherwise 0.
*dmy*_*multi*_*i*,*t*_	Dummy variable, 1 means that user *i* produced at least one multiple topics post in month *t*, otherwise 0.
*ln*⁡*culsingle*_*i*,*t*−1_	To the end of the month *t*−1, ln (1+ the total number of single topic posts posted by user *i*)
*ln*⁡*culmulti*_*i*,*t*−1_	To the end of the month *t*−1, ln (1+ the total number of multiple topics posts posted by user *i*)
*firm*_*fklsm*_*i*,*t*−1_	To the end of the month *t*−1, LSM of the content between all posts posted by user *i* and all firms’ feedback
*ptemo*_*i*,*t*−1_	To the end of the month *t*−1, the average emotional value of all posts posted by user *i*
*ln*⁡*ptlen*_*i*,*t*−1_	To the end of the month *t*−1, the total text length of all posts posted by user *i*
*ln*⁡*comt*_*given*_*i*,*t*−1_	To the end of the month *t*−1, ln (1+the total number of posts commented on other users by user *i*)
*ln*⁡*firm*_*fknum*_*i*,*t*−1_	To the end of the month *t*−1, the total number of feedback from all firms that user *i* received
*ln*⁡*firm*_*fklen*_*i*,*t*−1_	To the end of the month *t*−1, the total text length of feedback from all firms that user *i* received
*firm*_*emo*_*i*,*t*−1_	To the end of the month *t*−1, the average emotional value of feedback from all firms that user *i* received
*ln*⁡*user*_*fknum*_*i*,*t*−1_	To the end of the month *t*−1, the number of feedback that user *i* received from other users
*ln*⁡*user*_*fklen*_*i*,*t*−1_	To the end of the month *t*−1, total length of feedback text that user *i* received from other users
*user*_*fklsm*_*i*,*t*−1_	To the end of the month *t*−1, LSM of the content between all posts posted by user *i* and all other users’ feedback
*user*_*emo*_*i*,*t*−1_	To the end of the month *t*−1, the average amount of feedback received from other users of user *i*

The descriptive statistics and correlation analysis among variables are shown in Table 4. The results show that the difference between the mean and standard deviation of each variable is not very large, so there is no need to worry too much about the estimation bias caused by outliers.

**TABLE 4:** Descriptive statistics of variables.

Name of variables	Mean	SD	Min	Max
*dmy*_*single*_*i*,*t*_	0.119	0.324	0	1
*dmy*_*multi*_*i*,*t*_	0.081	0.273	0	1
*ln*⁡*culsingle*_*i*,*t*−1_	0.568	0.472	0	4.635
*ln*⁡*culmulti*_*i*,*t*−1_	0.369	0.45	0	4.564
*firm*_*fklsm*_*i*,*t*−1_	0.535	0.177	0.001	1
*ptemo*_*i*,*t*−1_	0.247	0.526	−1	1
*ln*⁡*ptlen*_*i*,*t*−1_	4.689	1.105	0.693	10.298
*ln*⁡*comt*_*given*_*i*,*t*−1_	0.478	0.894	0	7.182
*ln*⁡*firm*_*fknum*_*i*,*t*−1_	0.424	0.517	0	4.489
*ln*⁡*firm*_*fklen*_*i*,*t*−1_	4.12	1.029	1.099	8.592
*firm*_*emo*_*i*,*t*−1_	0.219	0.559	−1	1
*ln*⁡*user*_*fknum*_*i*,*t*−1_	1.673	0.992	0	6.94
*ln*⁡*user*_*fklen*_*i*,*t*−1_	4.847	1.388	0.693	10.524
*user*_*fklsm*_*i*,*t*−1_	0.526	0.169	0.001	1
*user*_*emo*_*i*,*t*−1_	0.232	0.408	−1	1

Furthermore, the correlation coefficient between variables preliminarily measures the problem of collinearity between variables. Table 5 shows the correlation coefficients of the main variables, and the correlation of all variables are shown in Supplementary Table 3. According to the results of the correlation table, the correlation coefficient between the main variable and other variables is not large, so it is reasonable to think that the problem of collinearity between variables is not serious. Especially, Table 5 shows that *dmy*_*single*_*i*,*t*_ and *dmy*_*multi*_*i*,*t*_ are weakly but significantly and not negatively correlated, which supports that both H1b and H2b are non-redundant.

**TABLE 5:** Correlation coefficient analysis of core variables.

Variable	(1)	(2)	(3)	(4)	(5)
*dmy*_*single*_*i*,*t*_	1.000				
*dmy*_*multi*_*i*,*t*_	0.107***	1.000			
*ln*⁡*culsingle*_*i*,*t*−1_	0.275***	−0.077***	1.000		
*ln*⁡*culmulti*_*i*,*t*−1_	−0.072***	0.350***	−0.177***	1.000	
*firm*_*fkl*s*m*_*i*,*t*−1_	−0.014**	−0.036***	−0.054***	−0.096***	1.000

**p < 0.05 and ***p < 0.01.

It is worth noting that, according to the sign and significance of correlation coefficient in Table 5, the number of posts with a single topic produced by users *ln*⁡*culsingle*_*i*,*t*−1_ is positively correlated with the *dmy*_*single*_*i*,*t*_. Similarly, the number of multiple topics posts produced by users *ln*⁡*culmulti*_*i*,*t*−1_ is positively correlated with the *dmy*_*multi*_*i*,*t*_ that whether users will produce multiple topics posts in the following month. In addition, *dmy*_*single*_*i*,*t*_ and*dmy*_*multi*_*i*,*t*_ have significant negative correlation with *firm*_*fkl*s*m*_*i*,*t*−1_. Although all the signs of these correlations are consistent with all hypothesis, the correlation is not causality. Therefore, this paper will verify the causality more rigorously in the following section.

## Results

Table 6 shows the regression results of the panel Logistics model, which confirm that users show an intrinsic variety-seeking tendency in producing UGCs.

**TABLE 6:** Panel Logistics model regression results.

	Model 1	Model 2
DV	*dmy*_*single*	*dmy*_*multi*
*ln*⁡*culsingle*	−3.880***	0.925***
	(0.342)	(0.289)
*ln*⁡*culmulti*	1.395***	−4.601***
	(0.253)	(0.358)
*firm*_*fklsm*	1.661***	3.257***
	(0.592)	(0.607)
*ptemo*	0.122	1.081***
	(0.330)	(0.362)
*ln*⁡*ptlen*	−0.141	−0.040
	(0.169)	(0.176)
*ln*⁡*comt*_*given*	0.539***	0.773***
	(0.125)	(0.144)
*ln*⁡*firm*_*fknum*	−0.114	1.140***
	(0.410)	(0.433)
*ln*⁡*firm*_*fklen*	−0.183	−0.562***
	(0.192)	(0.209)
*firm*_*fk*emo	−0.057	0.680**
	(0.256)	(0.302)
*ln*⁡*user*_*fknum*	−0.677**	−0.410
	(0.302)	(0.332)
*ln*⁡*user*_*fklen*	0.246	0.221
	(0.185)	(0.193)
*user*_*fklsm*	−0.494	−0.156
	(0.577)	(0.619)
*user*_*fk*emo	0.803**	0.036
	(0.372)	(0.410)
Individual FE	Yes	Yes
Time FE	Yes	Yes
N_sample	5,058	4,095
N_individuals	693	547

**p < 0.05, ***p < 0.01, SE in parentheses; FE means fix effect, the same below.

First, the cumulative number of posts on a single topic *ln*⁡*culsingle* has a significant negative effect on *dmy*_*single*, but a significant positive effect on *dmy*_*multi*. This means that if the user produced more single topic posts in total, the probability that users would produce single topic posts is lower and the probability that the user would produce multiple topics posts is higher in the following. Thus, H1a and H1b are supported.

Second, the cumulative number of multiple topics posts by users *ln*⁡*culmulti* has a significant positive impact on *dmy*_*single*, but a significantly negative impact on *dmy*_*multi*. Thus, if the user produced more multiple topics posts in total, the probability of producing single topic posts in the following time is higher, but the probability of producing multiple topics posts is lower. H2a and H2b are also supported.

Finally, *firm*_*fklsm* has a significantly positive effect on both *dmy*_*single* and *dmy*_*multi*. The results show that the closer the language style between firm feedbacks and the posts already produced by users, the higher the probability of producing both single topic posts and multiple topics posts, and both H3a and H3b are supported.

According to the above analysis, the higher the *firm*_*fklsm*, the higher the probability of producing both single topic post and multiple topics posts. This may seem contradictory, as users choose either a single topic or multiple topics in a UGC. However, users are not limited by the number of posts they can produce and *firm*_*fklsm* may encourage users to produce more posts. To demonstrate whether this could make sense, this study further analyzes the influence of *firm*_*fklsm* feedback on the number of posts per month and the results are shown in Table 7.

**TABLE 7:** The impact of LSM on the number of monthly posts by users (Threshold = 0.5).

	Model 3	Model 4	Model 5

	**Fixed effect model**	**Negative binominal model**	**Poisson model**
**DV**	***number of posts***	***number of posts***	***number of posts***
*ln*⁡*culsingle*	−0.158	−4.490***	1.187***
	(0.132)	(0.376)	(0.227)
*ln*⁡*culmulti*	−0.262***	0.921**	−2.676***
	(0.079)	(0.382)	(0.329)
*firm*_*fklsm*	1.715***	1.912***	2.317***
	(0.656)	(0.711)	(0.517)
*ptemo*	0.624	−0.198	0.779**
	(0.429)	(0.413)	(0.311)
*ln*⁡*ptlen*	0.190	−0.270	−0.154
	(0.128)	(0.225)	(0.150)
*ln*⁡*comt*_*given*	0.229**	0.501***	0.606***
	(0.094)	(0.171)	(0.120)
*ln*⁡*firm*_*fknum*	0.165	0.123	0.230
	(0.671)	(0.473)	(0.384)
*ln*⁡*firm*_*fklen*	−0.094	0.129	−0.499***
	(0.199)	(0.225)	(0.183)
*firm*_*fk*emo	0.346	0.026	0.149
	(0.304)	(0.345)	(0.241)
*ln*⁡*user*_*fknum*	−0.481***	−0.614	−0.997***
	(0.146)	(0.401)	(0.274)
*ln*⁡*user*_*fklen*	0.149**	0.280	0.387**
	(0.075)	(0.259)	(0.158)
*user*_*fklsm*	0.072	0.389	−0.165
	(0.293)	(0.728)	(0.505)
*user*_*fk*emo	−0.032	1.353***	0.101
	(0.134)	(0.481)	(0.322)
Individual FE	Yes	Yes	Yes
Time FE	Yes	Yes	Yes
N_sample	25,618	6,506	6,506
N_individuals	4,268	898	898

**p < 0.05, ***p < 0.01, SE in parentheses; FE means fix effect.

We used the panel fixed effects model at first, confirming that the higher *firm*_*fklsm* is, the higher the number of posts per month. H4 is supported. Second, because the number of posts is count data (non-negative integer), linear regression might be biased when count data is the dependent variable. Therefore, we used the panel negative binomial model and panel Poisson model, both of which are dedicated to count data, to test whether the promotion effect of firm feedback LSM on the number of posts per month still exists. All the corresponding results in Model 3–5 supported H4.

In conclusion, the LSM between firm feedbacks and UGCs will positively improve the probability of producing single topic and multiple topics posts, which will ultimately manifest as more posts will be produced.

### Robustness Check

Under the premise that the threshold defined by a single topic is 0.5, the results all supported the hypothesis proposed in this paper. However, we need to test whether the results remain the same if we change the threshold value. In this section, to prove the robustness of the results in this paper, the single topic threshold will be changed. We will change the threshold to 0.6 and 0.7 and define a post whose probability of belonging to any topic exceeds the threshold as a single topic post, multiple topics post otherwise. Regression results were shown in Table 8.

**TABLE 8:** Robustness test – regression results under different thresholds.

	Model 6	Model 7	Model 8	Model 9
threshold	0.6	0.6	0.7	0.7
DV	*dmy*_*single*	*dmy*_*multi*	*dmy*_*single*	*dmy*_*multi*
*ln*⁡*culsingle*	−4.201***	1.139***	−4.490***	1.187***
	(0.354)	(0.243)	(0.376)	(0.227)
*ln*⁡*culmulti*	1.464***	−3.520***	0.921**	−2.676***
	(0.325)	(0.336)	(0.382)	(0.329)
*firm*_*fklsm*	1.612***	2.671***	1.912***	2.317***
	(0.606)	(0.552)	(0.711)	(0.517)
*ptemo*	−0.105	0.996***	−0.198	0.779**
	(0.344)	(0.336)	(0.413)	(0.311)
*ln*⁡*ptlen*	−0.364*	0.086	−0.270	−0.154
	(0.192)	(0.157)	(0.225)	(0.150)
*ln*⁡*comt*_*given*	0.563***	0.638***	0.501***	0.606***
	(0.142)	(0.127)	(0.171)	(0.120)
*ln*⁡*firm*_*fknum*	0.201	0.273	0.123	0.230
	(0.430)	(0.405)	(0.473)	(0.384)
*ln*⁡*firm*_*fklen*	0.090	−0.500**	0.129	−0.499***
	(0.197)	(0.197)	(0.225)	(0.183)
*firm*_*fk*emo	0.174	0.194	0.026	0.149
	(0.285)	(0.257)	(0.345)	(0.241)
*ln*⁡*user*_*fknum*	−0.847**	−0.792***	−0.614	−0.997***
	(0.351)	(0.287)	(0.401)	(0.274)
*ln*⁡*user*_*fklen*	0.308	0.266	0.280	0.387**
	(0.215)	(0.168)	(0.259)	(0.158)
*user*_*fklsm*	−0.389	0.271	0.389	−0.165
	(0.631)	(0.534)	(0.728)	(0.505)
*user*_*fk*emo	0.914**	0.019	1.353***	0.101
	(0.407)	(0.357)	(0.481)	(0.322)
Individual FE	Yes	Yes	Yes	Yes
Time FE	Yes	Yes	Yes	Yes
N_sample	4,056	5,128	3,025	5,703
N_individuals	542	698	403	769

**p < 0.05, ***p < 0.01, SE in parentheses; FE means fix effect.

The results proved that all hypotheses were supported no matter whether the probability of defining a post as a single topic post with a threshold of 0.6 or 0.7, which was the same as the results above. Therefore, it is reasonable to conclude that our results are robust and insensitive to different thresholds. In addition, this paper also tested the promotion effect of *firm*_*fklsm* on the number of monthly posts of users under different thresholds of 0.6 and 0.7, and the results proved that the promotion effect was still significantly positive. The corresponding regression results are shown in Supplementary Tables 4, 5.

The purpose of this study is to examine the impact of firm feedback on the variety of UGCs. Therefore, it is necessary and would be better to relax the threshold value of defining a single topic to 0.4 which considers the probability combination of two topics in a post as a single topic, to prove that the conclusion has stronger robustness.

According to the results in Table 9, all hypotheses were supported again even if the threshold value of single topic posts is relaxed to 0.4, or even the “single topic” posts are tolerated to contain two topics. The above analysis showed that users have a strong variety-seeking inclination when producing UGCs, while *firm*_*fklsm* will promote users to produce both single topic and multiple topics UGC. In addition, this paper also tested the promotion effect of *firm*_*fklsm* on the number of users’ monthly posts under the threshold of 0.4, and the results proved that the promotion effect was also positive significantly. See Supplementary Table 6 for details of these specific regression results.

**TABLE 9:** Robustness test (Threshold = 0.4).

	Model 10	Model 11
DV	*dmy*_*single*	*dmy*_*multi*
*ln*⁡*culsingle*	−0.415***	0.216***
	(0.060)	(0.045)
*ln*⁡*culmulti*	0.659***	−1.883***
	(0.093)	(0.199)
*firm*_*fklsm*	2.315***	3.096***
	(0.547)	(0.929)
*ptemo*	0.477	1.237**
	(0.311)	(0.576)
*ln*⁡*ptlen*	−0.160	−0.023
	(0.145)	(0.255)
*ln*⁡*comt*_*given*	0.442***	0.585***
	(0.121)	(0.205)
*ln*⁡*firm*_*fknum*	−0.052	1.080*
	(0.379)	(0.589)
*ln*⁡*firm*_*fklen*	−0.169	−1.073***
	(0.181)	(0.309)
*firm*_*fk*emo	−0.087	0.655
	(0.258)	(0.458)
*ln*⁡*user*_*fknum*	−1.077***	−1.263***
	(0.278)	(0.482)
*ln*⁡*user*_*fklen*	0.266	0.805***
	(0.168)	(0.307)
*user*_*fklsm*	−0.030	0.628
	(0.519)	(0.925)
*user*_*fk*emo	0.518	−1.179*
	(0.346)	(0.673)
Individual FE	Yes	Yes
Time FE	Yes	Yes
N_sample	5,515	3,347
N_individuals	751	451

**p < 0.05, ***p < 0.01, SE in parentheses; FE means fix effect.

## Discussion

Based on variety-seeking theory and topic modeling, this study examines the reasons why UGCs in online communities are of great variety and discusses the role of firm feedback. According to the results, users will show an inherent preference for variety in UGCs production; that is, the more single topic UGCs produced in the past, the higher the probability of producing multiple topics UGCs and the lower the probability of single topic UGCs, and vice versa. Consequently, users will spontaneously enrich their content in online communities. In addition, the LSM between firm feedback and UGCs affects users’ variety-seeking tendencies in the production of UGC. This effect is reflected in the fact that the probability of producing multiple topics UGCs will increase, producing single topic UGCs will also increase, and users will eventually produce more UGCs. This study makes some contributions to theory, methodology, and practical implications for firms to manage UGCs in online communities.

### Contributions

The first contribution is theoretical, which expands the scope of application of the variety-seeking theory. Existing studies on variety-seeking mainly focus on whether people show variety-seeking tendencies when they are consumers (e.g., Mittelman et al., 2014; Carlson et al., 2015; Lee et al., 2020), while ignoring the fact that people may show variety-seeking tendency in production when they become producers. This study shows that people also show a variety-seeking tendency in production behavior, and thus expands the boundary of variety-seeking behavior, which is an extension of the variety-seeking theory.

The second contribution is methodological. This study used topic modeling in machine learning to measure variety-seeking behavior in UGCs production. Existing studies have proposed various methods to measure variety-seeking in the domain of traditional products; the common method is to select the number of brands that do not repeat in a certain number of products (Levav and Zhu, 2009; Huang et al., 2019). However, as UGCs are text, previous methods are difficult and inappropriate for measuring the variety of UGCs. The innovative use of the topic modeling in this study makes it possible to measure the variety-seeking in UGCs production, which is a methodological contribution.

### Practical Implications

Our findings give an empirical explanation to firms that aim to understand why UGCs are so varied. The reason is that users have an intrinsic preference for variety in producing UGCs; users are more likely to produce single topic UGCs if they produced more multiple topics UGCs in the past, and vice versa. Consequently, contents in online communities will be richer.

Furthermore, our findings can help firms manage or influence the variety of UGCs. Firms can improve the variety of UGCs in online communities by improving the LSM between firm feedback and UGCs, and users would be more likely to produce both single topic UGCs and multiple topics UGCs simultaneously; ultimately, users will produce more UGCs. Thus, when providing feedbacks to UGCs, firms can improve LSM between feedbacks and UGCs, which will promote the variety of UGCs in online communities.

### Limitations and Future Research

There are some limitations to this study, and these suggest the need for further research. First, the results of this study may be affected by differences across communities, and future studies can further investigate the heterogeneity of communities.

Second, to measure the variety of UGC content using other methods and further verify the conclusions of this study, future studies can also try to use other methods to extract the topics or meanings of UGC. For example, manual coding can be an option. Although manual coding costs time and money, its accuracy is undoubtedly very high.

Third, this study analyzes text, but UGCs have other formats, such as video and voice, both of which are becoming increasingly popular, which is reflected in the rapid rise and popularity of YouTube, Tik-Tok, and Instagram. Future studies can also try to analyze the variety-seeking behaviors of users on UGCs in the form of video, voice, etc., and explore the role of firm feedback.

Finally, we used intrinsic preference to explain variety-seeking in UGCs production, while some scholars noticed that external factors influence variety-seeking in consuming or buying. Future research can also study the impact of such external factors on users’ variety-seeking behavior in UGCs production.

## Data Availability Statement

The original contributions presented in the study are included in the article/Supplementary Material, further inquiries can be directed to the corresponding author/s.

## Author Contributions

WX contributed to conceptualization, investigation, writing, and visualization, review, and editing. YM and DL contributed to the substantial revision. YM successfully applied for the sponsorship of our research. SZ contributed to conceptualization, methodology, software, formal analysis, and writing. All authors contributed to the article and approved the submitted version.

## Conflict of Interest

The authors declare that the research was conducted in the absence of any commercial or financial relationships that could be construed as a potential conflict of interest.

## Publisher’s Note

All claims expressed in this article are solely those of the authors and do not necessarily represent those of their affiliated organizations, or those of the publisher, the editors and the reviewers. Any product that may be evaluated in this article, or claim that may be made by its manufacturer, is not guaranteed or endorsed by the publisher.

## Footnotes

1
www.miui.com

2 For more details about genism, see https://github.com/RaRe-Technologies/gensim.

3 This is a Chinese function word dictionary and its ISBN is 9787100032797.

4 For more details about jieba, see: https://github.com/fxsjy/jieba.
