# Supplementary material for: Why Are User-Generated Contents So Varied? An Explanation Based on Variety-Seeking Theory and Topic Modeling
Source: Front Psychol. 2022 Mar 10;13:808785. doi: 10.3389/fpsyg.2022.808785 (PMC8960714; doi:10.3389/fpsyg.2022.808785)
Supplement: Supplementary file 1 [file Data_Sheet_1.docx]

## Appendix

**Appendix 1: Topic modeling**

In order to explain the meaning of parameters involved in building a topic modeling in detail, this paper follows four steps to build a topic model.

**
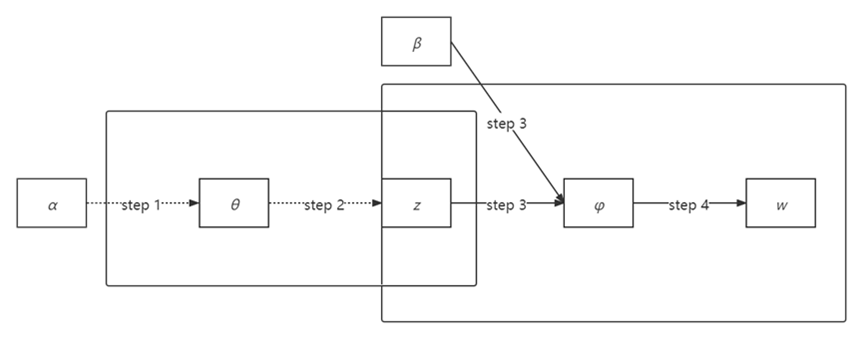
**Assuming that there are K topics in the text, the four steps of text $m$ generation can be further described as shown in the figure below.

**Figure 1** | Schematic diagram of the topic modeling construction process. where, the parameters α and β are the hyperparameters of Dirichlet Distribution, θ represents the topic distribution, $z$ represents the definite topic combination, $\varphi$ represents the word distribution, $w$ represents the definite word combination

**Step1:** determine the topic distribution $\theta_{m}$ of text $m$. In this process, it is assumed that $\theta_{m}$ is produced by a Dirichlet distribution with hyperparameter $\alpha$. And, the density function of Dirichlet distribution with random variable $\theta$ and hyperparameter $\alpha$ is:

$\boldsymbol{p}\left( \boldsymbol{\theta}_{\boldsymbol{m}}\boldsymbol{|\alpha} \right)\boldsymbol{=}\frac{\boldsymbol{1}}{\boldsymbol{\Delta}\left( \boldsymbol{\alpha} \right)}\prod_{\boldsymbol{i=1}}^{\boldsymbol{K}} \boldsymbol{\theta}_{\boldsymbol{m,i}}^{\boldsymbol{\alpha}_{\boldsymbol{i}}\boldsymbol{-1}}$ (1)

where，

$\boldsymbol{\Delta}\left( \boldsymbol{\alpha} \right)\boldsymbol{=}\frac{\prod_{\boldsymbol{i=1}}^{\boldsymbol{K}} \boldsymbol{\Gamma(}\boldsymbol{\alpha}_{\boldsymbol{i}}\boldsymbol{)}}{\boldsymbol{\Gamma(}\sum_{\boldsymbol{i=1}}^{\boldsymbol{K}} \boldsymbol{\alpha}_{\boldsymbol{i}}\boldsymbol{)}}\boldsymbol{,}\sum_{\boldsymbol{i=1}}^{\boldsymbol{K}} \boldsymbol{\theta}_{\boldsymbol{m,i}}\boldsymbol{=1}\boldsymbol{, \Gamma}\left( \boldsymbol{x} \right)\boldsymbol{=}\int\boldsymbol{t}^{\boldsymbol{x-1}}\boldsymbol{e}^{\boldsymbol{t}}\boldsymbol{dt}$ (2)

In equation (2) and below, $\Gamma$ denotes Gamma Distribution.

**Step 2:** according to the text topic distribution of $\theta_{m}$, determine the probability ($p\left( Z_{m}|\theta_{m} \right)$) of the K topics in text $m$, $Z_{m}$ is the topic set of the text $m$. So:

$\boldsymbol{p}\left( \boldsymbol{Z}_{\boldsymbol{m}}\boldsymbol{|}\boldsymbol{\theta}_{\boldsymbol{m}} \right)\boldsymbol{=}\prod_{\boldsymbol{i=1}}^{\boldsymbol{K}} \boldsymbol{\theta}_{\boldsymbol{m,i}}^{\boldsymbol{f}_{\boldsymbol{m}}^{\boldsymbol{(k)}}}$ (3)

Where $f_{m}^{(k)}$ represents the number of occurrences of the *k-th* topic in text $m$. Since the topic that determines one location of text $m$ is independent of the topic that determines the other location each time, $f_{m}$ will follow a polynomial distribution, that is:

$\boldsymbol{Multi}\left( \boldsymbol{f}_{\boldsymbol{m}}\boldsymbol{|}\boldsymbol{Z}_{\boldsymbol{m}}\boldsymbol{,N} \right)\boldsymbol{=}\binom{\boldsymbol{N}}{\boldsymbol{f}_{\boldsymbol{m}}}\prod_{\boldsymbol{i=1}}^{\boldsymbol{K}} {\boldsymbol{Z}_{\boldsymbol{m,i}}}^{\boldsymbol{f}_{\boldsymbol{m}}^{\boldsymbol{(i)}}}$ (4)

Where $Multi$ is polynomial distribution, and:

$\sum_{\boldsymbol{i=1}}^{\boldsymbol{K}} \boldsymbol{f}_{\boldsymbol{m}}^{\boldsymbol{(i)}}\boldsymbol{=N}\boldsymbol{,}\sum_{\boldsymbol{i=1}}^{\boldsymbol{K}} \boldsymbol{Z}_{\boldsymbol{m,k}}\boldsymbol{=1}\boldsymbol{,}\binom{\boldsymbol{N}}{\boldsymbol{f}_{\boldsymbol{m}}}\boldsymbol{=}\frac{\boldsymbol{N!}}{\prod_{\boldsymbol{i=1}}^{\boldsymbol{K}} \boldsymbol{f}_{\boldsymbol{m}}^{\boldsymbol{(i)}}}$ (5)

Combine Step 1 and step 2, we can draw $p_{m}\left( Z_{m}|\alpha\right)$ equals $p_{m}\left( \theta_{m}|\alpha\right)p\left( Z_{m}|\theta_{m} \right)$ on the subject distribution $\theta_{m}$ of integral in use of Bayesian formula, namely

$\boldsymbol{p}_{\boldsymbol{m}}\left( \boldsymbol{Z}_{\boldsymbol{m}}\boldsymbol{|\alpha} \right)\boldsymbol{=}\int\boldsymbol{p}_{\boldsymbol{m}}\left( \boldsymbol{\theta}_{\boldsymbol{m}}\boldsymbol{|\alpha} \right)\boldsymbol{p}\left( \boldsymbol{Z}_{\boldsymbol{m}}\boldsymbol{|}\boldsymbol{\theta}_{\boldsymbol{m}} \right)\boldsymbol{d}\boldsymbol{\theta}_{\boldsymbol{m}}$ (6)

Further, substitute equation (1) and equation (3) into equation (6) to obtain:

$\boldsymbol{p}_{\boldsymbol{m}}\left( \boldsymbol{Z}_{\boldsymbol{m}}|\boldsymbol{\alpha} \right)=\frac{\boldsymbol{1}}{\boldsymbol{\Delta}\left( \boldsymbol{\alpha} \right)}\int\prod_{\boldsymbol{i}=\boldsymbol{1}}^{\boldsymbol{K}} \boldsymbol{\theta}_{\boldsymbol{m},\boldsymbol{i}}^{\boldsymbol{\alpha}_{\boldsymbol{i}}+\boldsymbol{f}_{\boldsymbol{m}}^{\left( \boldsymbol{k} \right)}-\boldsymbol{1}}\boldsymbol{d}\boldsymbol{\theta}_{\boldsymbol{m}}$ (7)

Compared with equation (2), the integral part of equation (7) are different in parameters but the same in format. Therefore, equation (7) can be further written as:

$\boldsymbol{p}_{\boldsymbol{m}}\left( \boldsymbol{Z}_{\boldsymbol{m}}\boldsymbol{|\alpha} \right)\boldsymbol{=}\frac{\boldsymbol{\Delta}\left( \boldsymbol{\alpha+}\boldsymbol{f}_{\boldsymbol{m}} \right)}{\boldsymbol{\Delta}\left( \boldsymbol{\alpha} \right)}$ (8)

Up to now, the topic of text $m$ has been determined. Given the topic $Z_{m}$, next we will solve and determine the word $W_{m}$ of the text.

**Step 3**: according to the topic $Z_{m}$, assuming $V$ words in the dictionary of words on the topic of k obey distribution is $\varphi_{m,k} ={[\varphi}_{m,k}^{1},\varphi_{m,k}^{2},\ldots\ldots,\varphi_{m,k}^{V}]$, which is generated by the Dirichlet distribution with hyperparameter $\boldsymbol{\beta}$, namely:

$\boldsymbol{p}_{\boldsymbol{m}}\left( \boldsymbol{\varphi}_{\boldsymbol{m,k}}\boldsymbol{|\beta} \right)\boldsymbol{=}\frac{\boldsymbol{1}}{\boldsymbol{\Delta}\left( \boldsymbol{\beta} \right)}\prod_{\boldsymbol{t=1}}^{\boldsymbol{V}} \left( \boldsymbol{\varphi}_{\boldsymbol{m,k}}^{\boldsymbol{t}} \right)^{\boldsymbol{\beta}_{\boldsymbol{t}}\boldsymbol{-1}}$ (9)

**Step 4:** according to the word distribution $\varphi_{m,k}$, we can determine the probability of all the words that appear in topic k, namely to determine $p\left( w_{m,k}|\varphi_{m,k} \right)$, which is:

$\boldsymbol{p}\left( \boldsymbol{w}_{\boldsymbol{m,k}}\boldsymbol{|}\boldsymbol{\varphi}_{\boldsymbol{m,k}} \right)\boldsymbol{=}\prod_{\boldsymbol{t=1}}^{\boldsymbol{V}} \left( \boldsymbol{\varphi}_{\boldsymbol{m,k}}^{\boldsymbol{t}} \right)^{\boldsymbol{c}_{\boldsymbol{m,k}}^{\boldsymbol{(t)}}}$ (10)

Where, similar to Step 2,$c_{m,k}^{(t)}$ represents the frequency of occurrence of the t-th word in the total lexicon V of all texts in the *k-th* topic of text *m*, since the frequency of occurrence of any word in the *k-th* topic determined each time is independent of the frequency of occurrence of any other word. Therefore, $c_{m,k}$ will obey a polynomial distribution, and it is important to emphasize that there are N words in the text $m$, so:

$\boldsymbol{Multi}\left( \boldsymbol{c}_{\boldsymbol{m,k}}\boldsymbol{|}\boldsymbol{\varphi}_{\boldsymbol{m,k}}\boldsymbol{,N} \right)\boldsymbol{=}\binom{\boldsymbol{N}}{\boldsymbol{c}_{\boldsymbol{m,k}}}\prod_{\boldsymbol{t=1}}^{\boldsymbol{V}} \left( \boldsymbol{\varphi}_{\boldsymbol{m,k}}^{\boldsymbol{t}} \right)^{\boldsymbol{c}_{\boldsymbol{m,k}}^{\boldsymbol{(t)}}}$ (11)

where，

$\sum_{\boldsymbol{t=1}}^{\boldsymbol{V}} \boldsymbol{c}_{\boldsymbol{m,k}}^{\left( \boldsymbol{t} \right)}\boldsymbol{=N}\boldsymbol{,}\sum_{\boldsymbol{t=1}}^{\boldsymbol{V}} \boldsymbol{\varphi}_{\boldsymbol{m,k}}^{\boldsymbol{t}}\boldsymbol{=1}\boldsymbol{,}\binom{\boldsymbol{N}}{\boldsymbol{c}_{\boldsymbol{m,k}}}\boldsymbol{=}\frac{\boldsymbol{N!}}{\prod_{\boldsymbol{t=1}}^{\boldsymbol{V}} \boldsymbol{c}_{\boldsymbol{m,k}}^{\left( \boldsymbol{t} \right)}}$ (12)

Combination of step 3 and step 4, also using the Bayesian formula, we can conclude $\boldsymbol{p}_{\boldsymbol{m}}\left( \boldsymbol{w}_{\boldsymbol{m,k}}\boldsymbol{|\beta} \right)$equals $p_{m}\left( \varphi_{m,k}|\beta\right)$ and $p\left( w_{m,k}|\varphi_{m,k} \right)$ of the product on $\varphi_{m,k}$ integral, then there is:

$\boldsymbol{p}_{\boldsymbol{m}}\left( \boldsymbol{w}_{\boldsymbol{m,k}}\boldsymbol{|\beta} \right)\boldsymbol{=}\int\boldsymbol{p}_{\boldsymbol{m}}\left( \boldsymbol{\varphi}_{\boldsymbol{m,k}}\boldsymbol{|\beta} \right)\boldsymbol{p}\left( \boldsymbol{w}_{\boldsymbol{m,k}}\boldsymbol{|}\boldsymbol{\varphi}_{\boldsymbol{m,k}} \right)\boldsymbol{d}\boldsymbol{\varphi}_{\boldsymbol{m,k}}$ (13)

Substitute equation (9) and equation (10) into equation (13) to obtain:

$\boldsymbol{p}_{\boldsymbol{m}}\left( \boldsymbol{w}_{\boldsymbol{m,k}}\boldsymbol{|\beta} \right)\boldsymbol{=}\frac{\boldsymbol{1}}{\boldsymbol{\Delta}\left( \boldsymbol{\beta} \right)}\int\prod_{\boldsymbol{t=1}}^{\boldsymbol{V}} \left( \boldsymbol{\varphi}_{\boldsymbol{m,k}}^{\boldsymbol{t}} \right)^{\boldsymbol{\beta}_{\boldsymbol{t}}\boldsymbol{+}\boldsymbol{c}_{\boldsymbol{m,k}}^{\left( \boldsymbol{t} \right)}\boldsymbol{-1}}\boldsymbol{d}\boldsymbol{\varphi}_{\boldsymbol{m,k}}$ (14)

The integral part of equation (14) only has different parameters compared with equation (2). Therefore, equation (14) can be further written as:

$\boldsymbol{p}_{\boldsymbol{m}}\left( \boldsymbol{w}_{\boldsymbol{m,k}}\boldsymbol{|\beta} \right)\boldsymbol{=}\frac{\boldsymbol{\Delta}\left( \boldsymbol{\beta+}\boldsymbol{c}_{\boldsymbol{m,k}} \right)}{\boldsymbol{\Delta}\left( \boldsymbol{\beta} \right)}$ (14)

Since $K$ topics are independent of each other,

$\boldsymbol{p}_{\boldsymbol{m}}\left( \boldsymbol{W}_{\boldsymbol{m}}\boldsymbol{|}\boldsymbol{Z}_{\boldsymbol{m}}\boldsymbol{,\beta} \right)\boldsymbol{=}\prod_{\boldsymbol{i=1}}^{\boldsymbol{K}} \boldsymbol{p}_{\boldsymbol{m}}\left( \boldsymbol{w}_{\boldsymbol{m,k}}\boldsymbol{|\beta} \right)\boldsymbol{=}\prod_{\boldsymbol{i=1}}^{\boldsymbol{K}} \frac{\boldsymbol{\Delta}\left( \boldsymbol{\beta+}\boldsymbol{c}_{\boldsymbol{m,k}} \right)}{\boldsymbol{\Delta}\left( \boldsymbol{\beta} \right)}$ (15)

According to step1 to step4, the topic distribution and word distribution of text $m$ are finally generated. If again considering the bayesian formula, then for text m, the probability of its formation is $p_{m}\left( W_{m},Z_{m}|\alpha,\beta\right)=p_{m}\left( W_{m}|Z_{m},\beta\right)p_{m}\left( Z_{m}|\alpha\right)$, recall that the solution of $p_{m}\left( Z_{m}|\alpha\right)$ locates in the equations (8), the solution of $p_{m}\left( W_{m}|Z_{m},\beta\right)$ locates in equation (15), then:

$\boldsymbol{p}_{\boldsymbol{m}}\left( \boldsymbol{W}_{\boldsymbol{m}}\boldsymbol{,}\boldsymbol{Z}_{\boldsymbol{m}}\boldsymbol{|\alpha,\beta} \right)\boldsymbol{=}\frac{\boldsymbol{\Delta}\left( \boldsymbol{\alpha+}\boldsymbol{f}_{\boldsymbol{m}} \right)}{\boldsymbol{\Delta}\left( \boldsymbol{\alpha} \right)}\prod_{\boldsymbol{i=1}}^{\boldsymbol{K}} \frac{\boldsymbol{\Delta}\left( \boldsymbol{\beta+}\boldsymbol{c}_{\boldsymbol{m,k}} \right)}{\boldsymbol{\Delta}\left( \boldsymbol{\beta} \right)}$ (16)

Extend equation (16) to the text with a total of M, then we can get:

$\boldsymbol{p}\left( \boldsymbol{W,Z|\alpha,\beta} \right)\boldsymbol{=}\prod_{\boldsymbol{m=1}}^{\boldsymbol{M}} \frac{\boldsymbol{\Delta}\left( \boldsymbol{\alpha+}\boldsymbol{f}_{\boldsymbol{m}} \right)}{\boldsymbol{\Delta}\left( \boldsymbol{\alpha} \right)}\prod_{\boldsymbol{i=1}}^{\boldsymbol{K}} \frac{\boldsymbol{\Delta}\left( \boldsymbol{\beta+}\boldsymbol{c}_{\boldsymbol{k}} \right)}{\boldsymbol{\Delta}\left( \boldsymbol{\beta} \right)}$ (17)

Finally, this is the topic modeling we need.

For equation (17), the total number of texts M is known. The number of topics K needs to be set in advance, which can be judged according to experience or text discreteness corresponding to different topics (Kaplan and Vakili, 2015; Zhong and Schweidel, 2020). According to Griffiths and Steyvers (2004), $\alpha$ does not affect the number and relative importance of topics, that is, the topic with the highest probability of topics in a text is still the one with the highest probability after α transformation. Similarly, $\beta$ does not affect the relative importance of each word in the distribution of words under each topic, and the words with the highest occurrence probability in the distribution of words under each topic still have the highest occurrence probability under different $\beta$. Therefore, the default$\alpha$ and $\beta$ in the software will be adopted in the actual calculation in this paper, while $f_{m}\mathrm{and}c_{k}$ parameters can be solved by Gibbs Sampling.

**Reference**

Kaplan, S., and Vakili, K. (2015). The double-edged sword of recombination in breakthrough innovation. *Strateg. Manage. J.* 36(10), 1435-1457. doi: 10.1002/smj.2294.

Griffiths, T. L., and Steyvers, M. (2004). Finding scientific topics. *Proc. Nat. Acad. Sci.*101(suppl 1), 5228-5235. doi: 10.1073/pnas.0307752101.

Zhong, N., and Schweidel, D. A. (2020). Capturing changes in social media content: a multiple latent changepoint topic model. *Mark. Sci.*39(4), 827-846. doi: 10.1287/mksc.2019.1212.

**Appendix 2: Supplementary Figures and Tables**

**Table A1** | Table A1 The top 20 words with the highest frequency in 11 topics

| Topic | The top 20 words | Explanation on Topic |
| --- | --- | --- |
| Topic 1 | [' functions', 'messages',' recommendations', 'in ',' hope ', 'has',' add ', 'process', 'reply', 'software', 'delete', 'desktop', 'very', 'application', 'block', 'no', 'notify', 'set up', 'join', 'all'] | MIUI SMS function related |
| Topic 2 | [' topic ',' application ', 'MIUI', 'Xiaomi', 'all', 'no', 'system', 'user', 'updated', 'very', 'function', 'good', 'hope', 'shop', 'mobile phone', 'development', 'said'， 'now'，'modules',' miui'] | MIUI topic related |
| Topic 3 | [' flow '，' show ', 'card', 'network', 'weather' ,'recommendations',' when ', '3G', 'mobile phone', 'Mobile' and 'Unicom', 'no', 'assistant', 'use', 'color', 'all', 'operator' , 'package', 'hope' , 'charging'] | Mobile plans, etc. |
| Topic 4 | [' functions', 'notify', 'phone', 'no', 'mobile phone', 'down', 'all', 'MIUI', 'will', 'number', 'on', 'bar', 'development', 'miui', 'screen', 'a', 'recommendations',' hope ', 'user', 'show'] | Notification function |
| Topic 5 | [' mobile phone ', 'millet', 'cloud', 'function', 'service' and 'sync', 'backup', 'on', 'all', 'messages',' hope ', 'no', 'contacts,' not 'and' no ', 'computer', 'use', 'recommendations',' download 'and' after '] | Communication cloud services |
| Topic 6 | [' is', 'no', 'video', 'Xiaomi', 'function', 'hope', 'not' and 'system', 'problem', 'own', 'power', 'support', 'recognition', 'development', 'no', 'player', 'a', 'beautiful MIUI', 'music' and 'font'] | Audio features |
| Topic 7 | [' mobile phone ', 'no', 'all', 'password', ' Xiaomi ', 'no', 'know', 'updated', 'system', 'input', 'problem', 'now,' want to ', 'version', 'not', 'people', 'no', 'said', 'and', 'function'] | System update problem |
| Topic 8 | [' icon ', 'show', 'desktop', 'no', 'contacts',' set up ', 'all', 'status bar', 'hidden', 'a', 'interface', 'no', 'search', 'animated', 'hope' and 'will' and 'application', 'not', 'mode', 'digital'] | Desktop function |
| Topic 9 | [' model 'and' image ', 'safe', 'center', 'album', 'gallery', 'application', 'recommendations',' photos', 'file', 'set up', ' ', 'camera', 'no', 'after', 'unlock', 'hope', 'system', 'visitors' and' function '] | Cameras and security services |
| Topic 10 | [' functions', 'calendar', 'music', 'hope', 'remind', 'set up', 'all', 'a', 'ring', 'not' and 'volume', 'headphone', 'Xiaomi,' mobile phone ', 'no', 'development', 'voice', 'mode', 'the', 'now'] | Sound function correlation |
| Topic 11 | [' no ', 'automatic', 'set up', 'mode', 'shut down', 'alarm', 'will', 'screen', 'network', 'after', 'use', 'all', 'when', 'mobile phone', 'time', 'open', 'to', 'functions',' open 'and' switch '] | Switch function |

**Table A2 |** Examples of posts corresponding to the 11 topics

| Topic | Explanation on Topic | The Original Post | Probability of the Topic | Link |
| --- | --- | --- | --- | --- |
| Topic 1 | MIUI SMS function related | - *"I use the contact group function in MIUI, which is very helpful for contact classification. But now I have a hope. Such as my group has: \| "Shanghai" \| "Harbin" \| \| "students" "teacher" \| "high school" \| \| "university"......"* | 0.982 | http://www.miui.com/thread-1331339-1-1.html |
| Topic 2 | MIUI topic related | - *"Damn it, now the default font is applied to all themes. The result will be restored to the default font. Reset the font every time you apply the theme. Except for the font. A lot of times. Themes are unlikely to be perfect in every module. Every module that can't be done is liked by users. So it's recommended to apply themes. ..."* | 0.989 | http://www.miui.com/thread-1686014-1-1.html |
| Topic 3 | Mobile plans, etc. | - *"Send between 2082 and 10010, received the following reply \| \| \| as of October 7, your meal allowance for the month information is as follows: ... Now the 2G card of Ningxia Unicom cannot be automatically corrected with traffic. I hope it can be repaired."* | 0.968 | http://www.miui.com/thread-1426029-1-1.html |
| Topic 4 | Notification function | - *"It is suggested to add in the notification bar Settings can choose to slide in any position on the desktop gesture can pull down the notification bar, I am a one meter eight boy, one hand control, pull down the notification bar are slightly difficult, in order to facilitate operation, it is suggested to add this empty. ADW desktop has this function, very humane. \| \| I searched, this function has included a year ago. \| evidence: http://www.miui.com/thread-627836-1-1.html \| \| but, why haven't solve? \| doesn't mean nothing to the harms. ~!!!!!!!!!!"* | 0.981 | http://www.miui.com/thread-1392112-1-1.html |
| Topic 5 | Communication cloud services | - *"Cloud services to retrieve the phone is why the location of the phone number, rather than an allele phone IMEI string number. I think the user should be able to locate and control the encryption of the phone or delete data, take photos and so on when the phone is connected to the network or GPS. \| find existing mobile phone function is not practical, \| usually someone pick up to or theft to the phone will clear data first, and clear data back again after the mobile phone is almost completely impossible, \| if the way is to use the find phone will be very useful, \| specific methods such as millet company through the IMEI and millet mobile phone after the certification report to monitor the phone by millet company/positioning, and notify the user to help users find mobile phone, \| fix this later believe there will be more users choose millet mobile phone \| \| want beautiful MIUI team adopted \|"* | 0.991 | http://www.miui.com/thread-1322512-1-1.html |
| Topic 6 | Audio features | - *"Xiaomi built-in music player is my favorite player, but not such a good player supports WMA format music, \| WMA format according to the code in the music player, this is a very egg pain, hope that the official to improve. \| \| -- -- -- -- -- - support Xiaomi"* | 0.972 | http://www.miui.com/thread-1253877-1-1.html |
| Topic 7 | System update problem | - *"How now I download the latest version of the system MIUI JLB21.0 stable version can’t see the privacy of the message, ask the master to help ah, how to do, before useful privacy message, now update the system can’t find, kneeling solution"* | 0.965 | http://www.miui.com/thread-1360705-1-1.html |
| Topic 8 | Desktop function | - *"This post by LJXHJD finally in the 2013-6-15 good tidings to edit \| \| can develop a like IOS7 weibos as background??? So the backstage is beautiful!! There is also in unlock after can like IOS7 have an animation effect?? And can the search bar be hidden like IOS7, and when you go back to the desktop from an app the animation is not clear can you make it like IOS7 from an app back to the desktop animation is a little bit more obvious."* | 0.976 | http://www.miui.com/thread-1250180-1-1.html |
| Topic 9 | Cameras and security services | - *"This is not a bug or defect For example I set gallery for privacy protection into the gallery when I need to unlock But I point from the camera I don't need to unlock Although it is photo album But I don't want others to see this can adjust the \| another problem is hope team can adjust the automatic shutdown of the power limit Because if accidentally shut down automatically press the power button will have no feedback and broken, can you leave a little power automatically shut down and then press the power button can make the phone light is also ok."* | 0.984 | http://www.miui.com/thread-1282682-1-1.html |
| Topic 10 | Sound function correlation | - *"Please set the ring tone for 2S development version to crescendo!! \| 2S development version of the phone ring, please set the bell gradually strong!!!!!! \| 2S development version of the phone ring, please set the bell gradually strong!!!!!! \| 2S development version of the phone ring, please set the bell gradually strong!!!!!! \| 2S development version of the phone ring, please set the bell gradually strong!!!!!! \| 2S development version of the phone ring, please set the bell gradually strong!!!!!!!!!"* | 0.985 | http://www.miui.com/thread-1319439-1-1.html |
| Topic 11 | Switch function | - *"Millet alarm clock personal feel very good, but also can be better, I have a suggestion, if I rest tomorrow, today will shut the alarm clock before he goes to bed at night, if I forget to open the alarm clock tomorrow, the day after tomorrow to work the alarm clock ring, may be late for me because it has been late for several times so I think, if the alarm clock can suspend, good, Alarm can choose Saturday and Sunday don't go off now, it will ring on Monday morning, but if I ask for leave on Wednesday, a day, that I first off the alarm clock on Tuesday night, the alarm clock ring on Wednesday, can have a good sleep, but if I forget to open the alarm clock on Wednesday night, that I might be late for the alarm clock rang on Thursday, so I suggest that add a pause to the alarm clock function, if I ask for leave on Wednesday, Wouldn't it be easier to pause for the day on or before Tuesday night and have your alarm clock go off on Wednesday and resume automatically on Thursday morning??* | 0.989 | http://www.miui.com/thread-1253097-1-1.html |

**Table A3** | Correlation coefficient analysis of all variables

| variables | (1) | (2) | (3) | (4) | (5) | (6) | (7) | (8) | (9) | (10) | (11) | (12) | (13) | (14) |
| --- | --- | --- | --- | --- | --- | --- | --- | --- | --- | --- | --- | --- | --- | --- |
| $dmy\_s{ingle}_{i,t}$ | - |  |  |  |  |  |  |  |  |  |  |  |  |  |
| $dmy\_multi_{i,t}$ | 0.107* | - |  |  |  |  |  |  |  |  |  |  |  |  |
| $\ln culsingle_{i,t-1}$ | 0.275* | -0.077* | - |  |  |  |  |  |  |  |  |  |  |  |
| $\ln culmulti_{i,t-1}$ | -0.072* | 0.350* | -0.177* | - |  |  |  |  |  |  |  |  |  |  |
| $firm\_fklsm_{i,t-1}$ | -0.014 | -0.036* | -0.054* | -0.096* | - |  |  |  |  |  |  |  |  |  |
| ${ptemo}_{i,t-1}$ | -0.005 | 0.017* | -0.021* | 0.037* | -0.019* | - |  |  |  |  |  |  |  |  |
| $\ln{ptlen}_{i,t-1}$ | 0.032* | 0.157* | 0.203* | 0.482* | -0.241* | 0.060* | - |  |  |  |  |  |  |  |
| $\ln{comt\_given}_{i,t-1}$ | 0.069* | 0.092* | 0.321* | 0.294* | -0.077* | 0.017* | 0.311* | - |  |  |  |  |  |  |
| $\ln{firm\_fknum}_{i,t-1}$ | 0.086* | 0.101* | 0.398* | 0.350* | 0.007 | -0.004 | 0.341* | 0.285* | - |  |  |  |  |  |
| $\ln{firm\_fklen}_{i,t-1}$ | 0.053* | 0.099* | 0.300* | 0.332* | 0.073* | -0.028* | 0.437* | 0.266* | 0.625* | - |  |  |  |  |
| ${firm\_emo}_{i,t-1}$ | 0.008 | -0.004 | 0.035* | -0.011 | -0.033* | 0.089* | 0.017* | 0.023* | -0.017* | -0.039* | - |  |  |  |
| $\ln{user\_fknum}_{i,t-1}$ | 0.038* | 0.072* | 0.379* | 0.349* | -0.143* | 0.019* | 0.434* | 0.411* | 0.326* | 0.392* | 0.024* | - |  |  |
| $\ln{user\_fklen}_{i,t-1}$ | 0.046* | 0.085* | 0.325* | 0.322* | -0.118* | 0.002 | 0.453* | 0.369* | 0.329* | 0.432* | 0.010 | 0.848* | - |  |
| $user\_fklsm_{i,t-1}$ | 0.025* | 0.011* | 0.061* | 0.024* | 0.156* | -0.019* | -0.037* | 0.019* | 0.037* | 0.006 | -0.025* | -0.040* | -0.029* | - |
| ${user\_emo}_{i,t-1}$ | -0.002 | 0.005 | 0.009 | 0.018* | -0.012 | 0.139* | 0.048* | 0.026* | 0.020* | -0.037* | 0.122* | 0.022* | -0.018* | -0.022* |

**Table A4** | Influence of LSM on the number of monthly posts of users (threshold = 0.6)

|  | Model 3 | Model 4 | Model 5 |
| --- | --- | --- | --- |
| Model setting | Fixed effect model | Negative Binominal model | Poisson model |
| DV | $number of posts$ | $number of posts$ | $number of posts$ |
| $\ln culsingle$ | -1.264*** | -0.577** | -0.604*** |
|  | (0.329) | (0.246) | (0.135) |
| $\ln culmulti$ | -0.729*** | 0.001 | 0.124 |
|  | (0.275) | (0.185) | (0.101) |
| $firm\_fklsm$ | 0.998** | 1.105** | 1.216*** |
|  | (0.459) | (0.437) | (0.251) |
| $ptemo$ | 0.586* | 0.585* | 0.195 |
|  | (0.335) | (0.330) | (0.142) |
| $\ln ptlen$ | 0.324* | 0.059 | -0.081 |
|  | (0.188) | (0.146) | (0.070) |
| $\ln comt\_given$ | 0.141** | 0.293*** | 0.331*** |
|  | (0.071) | (0.104) | (0.047) |
| $\ln firm\_fknum$ | -0.813* | 0.132 | 0.413** |
|  | (0.455) | (0.320) | (0.164) |
| $\ln firm\_fklen$ | 0.212 | -0.036 | -0.253*** |
|  | (0.184) | (0.147) | (0.078) |
| $firm\_fk\mathrm{emo}$ | 0.295 | 0.359 | 0.039 |
|  | (0.241) | (0.252) | (0.120) |
| $\ln user\_fknum$ | -0.380** | -0.857*** | -0.535*** |
|  | (0.158) | (0.243) | (0.139) |
| $\ln user\_fklen$ | 0.198*** | 0.346** | 0.151* |
|  | (0.075) | (0.156) | (0.089) |
| $user\_fklsm$ | -0.038 | 0.505 | 0.242 |
|  | (0.296) | (0.480) | (0.271) |
| $user\_fk\mathrm{emo}$ | 0.011 | -0.038 | 0.238 |
|  | (0.153) | (0.300) | (0.175) |
| Individual FE | Yes | Yes | Yes |
| Time FE | Yes | Yes | Yes |
| N_sample | 25,618 | 6,506 | 6,506 |
| N_individual | 4,268 | 898 | 898 |

**Table A5** | Influence of LSM on the number of monthly posts of users (threshold = 0.7)

|  | Model 3 | Model 4 | Model 5 |
| --- | --- | --- | --- |
| Model setting | Fixed effect model | Negative Binominal model | Poisson model |
| DV | $number of posts$ | $number of posts$ | $number of posts$ |
| $\ln culsingle$ | -0.793** | -0.004 | 0.022 |
|  | (0.337) | (0.187) | (0.094) |
| $\ln culmulti$ | -1.033*** | -0.280 | -0.320** |
|  | (0.332) | (0.289) | (0.132) |
| $firm\_fklsm$ | 0.982** | 1.217*** | 1.347*** |
|  | (0.467) | (0.414) | (0.249) |
| $ptemo$ | 0.567* | 0.570* | 0.222 |
|  | (0.338) | (0.328) | (0.142) |
| $\ln ptlen$ | 0.317 | 0.018 | -0.065 |
|  | (0.201) | (0.158) | (0.071) |
| $\ln comt\_given$ | 0.116 | 0.285*** | 0.332*** |
|  | (0.073) | (0.103) | (0.047) |
| $\ln firm\_fknum$ | -0.897** | -0.031 | 0.293* |
|  | (0.433) | (0.315) | (0.166) |
| $\ln firm\_fklen$ | 0.226 | -0.014 | -0.259*** |
|  | (0.181) | (0.145) | (0.079) |
| $firm\_fk\mathrm{emo}$ | 0.297 | 0.369 | 0.027 |
|  | (0.241) | (0.252) | (0.120) |
| $\ln user\_fknum$ | -0.437*** | -0.970*** | -0.615*** |
|  | (0.162) | (0.262) | (0.139) |
| $\ln user\_fklen$ | 0.208*** | 0.380** | 0.174* |
|  | (0.074) | (0.158) | (0.090) |
| $user\_fklsm$ | -0.061 | 0.481 | 0.271 |
|  | (0.308) | (0.470) | (0.271) |
| $user\_fk\mathrm{emo}$ | -0.017 | -0.016 | 0.187 |
|  | (0.155) | (0.299) | (0.176) |
| Individual FE | Yes | Yes | Yes |
| Time FE | Yes | Yes | Yes |
| N_sample | 25,618 | 6,506 | 6,506 |
| N_individual | 4,268 | 898 | 898 |

**Table A6** | Influence of LSM on the number of monthly posts of users (threshold = 0.4)

|  | Model 3 | Model 4 | Model 5 |
| --- | --- | --- | --- |
| Model setting | Fixed effect model | Negative Binominal model | Poisson model |
| DV | $number of posts$ | $number of posts$ | $number of posts$ |
| $\ln culsingle$ | -1.264*** | -0.577** | -0.604*** |
|  | (0.329) | (0.246) | (0.135) |
| $\ln culmulti$ | -0.729*** | 0.001 | 0.124 |
|  | (0.275) | (0.185) | (0.101) |
| $firm\_fklsm$ | 0.998** | 1.105** | 1.216*** |
|  | (0.459) | (0.437) | (0.251) |
| $ptemo$ | 0.586* | 0.585* | 0.195 |
|  | (0.335) | (0.330) | (0.142) |
| $\ln ptlen$ | 0.324* | 0.059 | -0.081 |
|  | (0.188) | (0.146) | (0.070) |
| $\ln comt\_given$ | 0.141** | 0.293*** | 0.331*** |
|  | (0.071) | (0.104) | (0.047) |
| $\ln firm\_fknum$ | -0.813* | 0.132 | 0.413** |
|  | (0.455) | (0.320) | (0.164) |
| $\ln firm\_fklen$ | 0.212 | -0.036 | -0.253*** |
|  | (0.184) | (0.147) | (0.078) |
| $firm\_fk\mathrm{emo}$ | 0.295 | 0.359 | 0.039 |
|  | (0.241) | (0.252) | (0.120) |
| $\ln user\_fknum$ | -0.380** | -0.857*** | -0.535*** |
|  | (0.158) | (0.243) | (0.139) |
| $\ln user\_fklen$ | 0.198*** | 0.346** | 0.151* |
|  | (0.075) | (0.156) | (0.089) |
| $user\_fklsm$ | -0.038 | 0.505 | 0.242 |
|  | (0.296) | (0.480) | (0.271) |
| $user\_fk\mathrm{emo}$ | 0.011 | -0.038 | 0.238 |
|  | (0.153) | (0.300) | (0.175) |
| Individual FE | Yes | Yes | Yes |
| Time FE | Yes | Yes | Yes |
| N_sample | 25,618 | 4,966 | 4,966 |
| N_individual | 4,268 | 679 | 679 |
